# In Silico analysis of Gastric carcinoma Serial Analysis of Gene Expression libraries reveals different profiles associated with ethnicity

**DOI:** 10.1186/1476-4598-7-22

**Published:** 2008-02-27

**Authors:** Francisco J Ossandon, Cynthia Villarroel, Francisco Aguayo, Eudocia Santibanez, Naohide Oue, Wataru Yasui, Alejandro H Corvalan

**Affiliations:** 1 Laboratory of Molecular Pathology and Epidemiology, Centro Investigaciones Medicas, Pontificia Universidad Catolica de Chile, Santiago, Chile; 2 Department of Molecular Pathology, Hiroshima University, Graduate School of Biomedical Sciences, Hiroshima, Japan; 3 Department of Pathology, University of Texas, M.D. Anderson Cancer Center, Houston, TX 77030, USA

## Abstract

Worldwide gastric carcinoma has marked geographical variations and worse outcome in patients from the West compared to the East. Although these differences has been explained by better diagnostic criteria, improved staging methods and more radical surgery, emerging evidence supports the concept that gene expression differences associated to ethnicity might contribute to this disparate outcome. Here, we collected datasets from 4 normal and 11 gastric carcinoma Serial Gene Expression Analysis (SAGE) libraries from two different ethnicities. All normal SAGE libraries as well as 7 tumor libraries were from the West and 4 tumor libraries were from the East. These datasets we compare by Correspondence Analysis and Support Tree analysis and specific differences in tags expression were identified by Significance Analysis for Microarray. Tags to gene assignments were performed by CGAP-SAGE Genie or TAGmapper. The analysis of global transcriptome shows a clear separation between normal and tumor libraries with 90 tags differentially expressed. A clear separation was also found between the West and the East tumor libraries with 54 tags differentially expressed. Tags to gene assignments identified 15 genes, 5 of them with significant higher expression in the West libraries in comparison to the East libraries. qRT-PCR in cell lines from west and east origin confirmed these differences. Interestingly, two of these genes have been associated to aggressiveness (COL1A1 and KLK10). In conclusion we found that in silico analysis of SAGE libraries from two different ethnicities reveal differences in gene expression profile. These expression differences might contribute to explain the disparate outcome between the West and the East.

## Introduction

Gastric carcinoma is the second leading cause of cancer-related death worldwide and has marked geographical variations [1-3]. The observed advantage in 5-year survival rate from patients from the East than from the West may reflect differences in diagnostic criteria, better staging methods and more radical surgery [4]. However emerging evidence supports the concept that ethnicity might contribute to the disparate gastric carcinoma outcomes between the East and the West [4,5]. Serial Analysis of Gene Expression (SAGE) is a comprehensive profiling method that allows for global, unbiased and quantitative characterization of transcriptomes [6]. A major advantage of SAGE is that once normalized is possible to directly compare the levels of tags generated by a single experiment with any other available [7]. To gain an insight of the differences between gastric carcinoma transcriptomes that might explain the disparate outcomes between the East and the West here we compare datasets of fifteen SAGE libraries derived from normal and gastric tumor tissues from Japanese and American gastric cancer patients by Correspondence Analysis, Support Tree and Significance Analysis for Microarray for significative tags and gene selection. We found specific genes differentially expressed between normal and tumor SAGE libraries as well as tumor libraries from the East and the West. These differentially expressed genes could explain the worse survival rate in the West in comparison to the East.

## Methods

### Serial Analyses of Gene Expression data

Fifteen gastric SAGE libraries (4 normal and 11 tumor) from Cancer Genome Anatomy Project (CGAP) [7] were combined for the analysis. Only libraries with 10 bp tags and the same cutting enzymes (BsmFI and NlaIII) were included in this study. Normal libraries consist of a tissue pool (GSM784 and GSM14780) or microdissected samples (CGAP_MD_13S and CGAP_MD_14S) and were produced by El-Rifai et al [8] in Virginia, USA. Gastric tumor libraries consist of five libraries, three microdissected (CGAP_MD_HG7, CGAP_MD_HS29, CGAP_MD_G329), two primary tumors (GSM757 and GSM2385) and two xenografts (GSM758 and GSM14760) all from western patients and produced by El-Rifai et al [8] also in Virginia, USA ("West tumor libraries") and 4 libraries (GSM7800, GSM8505, GSM8867 and GSM9103) all from japanese patients produced by Oue et al [9] in Hiroshima, Japan ("East tumor libraries"). A database containing 121,409 different tags was generated from libraries which have between 9,000 and 34,000 unique tags. Thus, only library GSM9103 was removed because its unique tag count was too low (around 6,000 unique tags). The frequency of each tag was normalized by dividing it with the total tag number of the corresponding library and multiplying by 200,000 tags (CGAP normalization format). A selection process to reduce noise from an enormous amount of tags collected was performed. This selection criterion was i) "tags found in *all normal libraries*" vs. "tags found in *all tumor libraries*" and ii) "tags found in *all West tumors libraries*" vs. "tags found in *all East tumors libraries*". The Institute for Genomic Research software MultiExperiment Viewer [10] was used to perform the following analysis: i) Correspondence Analysis (COA) to explore associations between samples that tend to have similar profiles ii) Support Tree to shows the statistical support after repeating at least 1000 times the analysis by resampling with replacement (Bootstrap method) for samples with similar profiles and iii) Significance Analysis for Microarray (SAM) to select tags whose expression was significantly different between samples. The association of tags to genes was perform by SAGE Genie [11] or TAGmapper [12] when no association was found by SAGE Genie. To predict functional classes of annotated genes the FatiGO+ tool of Babelomics [13,14] was applied. The unadjusted p-value given by Babelomics was used because the small number of genes analyzed made it more appropriate than the adjusted-False Discovery Rate (FDR) value.

### Quantitative Real-Time Reverse-Transcription PCR

Quantitative real-time reverse-transcription PCR (qRT-PCR) was performed on two western cell lines (AGS, N87) and one eastern cell line (MKN45). Total RNA was extracted using Trizol (Invitrogen Life Technologies, Carlsbad, CA) according to the manufacturer's recommendations. RNA concentration was determined by measuring absorbance at 260 nm, and quality was verified by the integrity of 28S and 18S rRNA after ethidium bromide staining of total RNA samples subjected to 0.8% agarose gel electrophoresis. Total cDNA was synthesized with MMLV (Moloney Murine Leukemia Virus) reverse transcriptase (ThermoScript RT; Invitrogen Life Technologies, Carlsbad, CA). Reverse transcription-PCR was performed using 1 ug of total cellular RNA to generate cDNA. qRT-PCR was performed using a LightCycler-FastStart DNA Master SYBR Green I kit (Roche Molecular Biochemicals, Mannheim, Germany). We designed gene-specific primers for human PDFGR (5' AGCTGATCCGTGCTAAGGAA 3' and 5' CGACCAAGTCCAGAATGGAT 3') and RPL13 (5' GAGGAGGCGGAACAAGTCC 3' and 5' TCAGCAGAACTGTCTCCCTTC 3') and conditions of amplification are available upon request. A single-melt curve peak was observed for each product, thus confirming the purity of all amplified cDNA products. The qRT-PCR results were normalized to GADPH (5' CGGGAAGCTTGTCATCAATGG 3' and 5' CATGGTTCACACCCATGACG 3'), which had minimal variation in all cell lines tested. Analysis was performing by LightCycler software 3.0. Crossing points (beginning of the PCR exponential phase) were assessed by the second derivated maximum method and plotted against the concentrations of the standards.

## Results

### Tags with consistent expression in normal and tumor SAGE libraries

**Figure 1:**
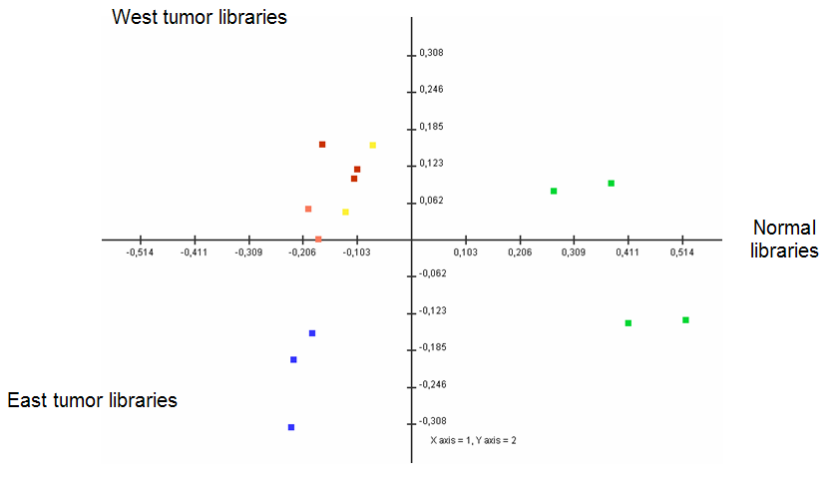
Correspondence Analysis of normal and tumor SAGE libraries of the stomach. A two-dimensional plot is shown where the green dots represent all the normal libraries, the blue dots are the East tumor libraries, and the red, orange and yellow dots are West tumor libraries, microdissected, xenograft and bulk respectively.

**Figure 2:**
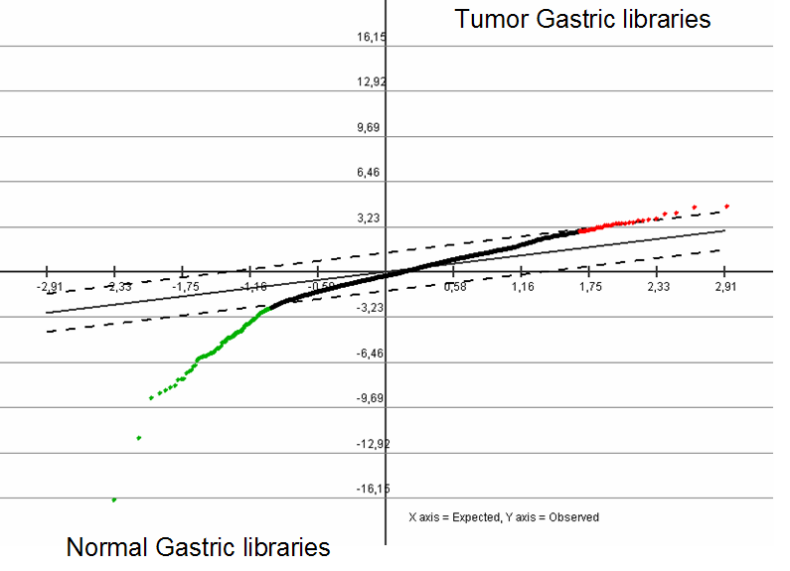
Serial Analysis for Microarray of normal and tumor SAGE libraries of the stomach. To the left and shown in green color, the significant tags with higher expression in the normal libraries; to the right and shown in red color, the significant tags with higher expression in the tumor libraries.

The selection process to find SAGE tags that were consistently expressed in "*all normal libraries*" vs. "*all tumor libraries*" resulted in 2,437 tags. As shown in Fig. 1, COA shows clear separation between normal libraries and East and West tumor libraries. The same COA in a three-dimensional plot (accounting for 56% of the total inertia) shows more details in the position of each library (see Additional File 1). These results were confirmed by a Support Tree using the Pearson Correlation and Average Linkage (see Additional File 2). Next, to identify SAGE tags differentially expressed between normal and tumor samples, we performed SAM, with a delta value of 1.38 calculated to maintain the FDR near to 0 (probability to find significant tags merely by chance), 1001 unique permutations and a fold change = 10. This approach revealed 90 tags differentially expressed between normal and tumor libraries with a similar behavior for both tumor groups (Fig. 2). Among these 90 tags, 78 were down-regulated and 12 tags were up-regulated.

### Selection of discriminatory tags between East and West SAGE libraries

**Figure 3:**
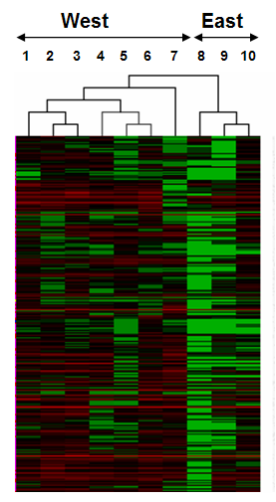
Support Tree of normal and tumor SAGE libraries of the stomach. Lanes 1–4 normal libraries (CGAP_MD_13S, GSM784, CGAP_MD_14S, GSM14780), lanes 5–11 West tumor libraries (CGAP_MD_HG7, CGAP_MD_HS29, CGAP_MD_G329, GSM757, GSM758, GSM14760, GSM2385) and lanes 12–14 East tumor libraries (GSM7800, GSM8505, and GSM8867). Only the top of the dendrogram is shown here. The full dendrogram appear in Additional File 3.

**Figure 4:**
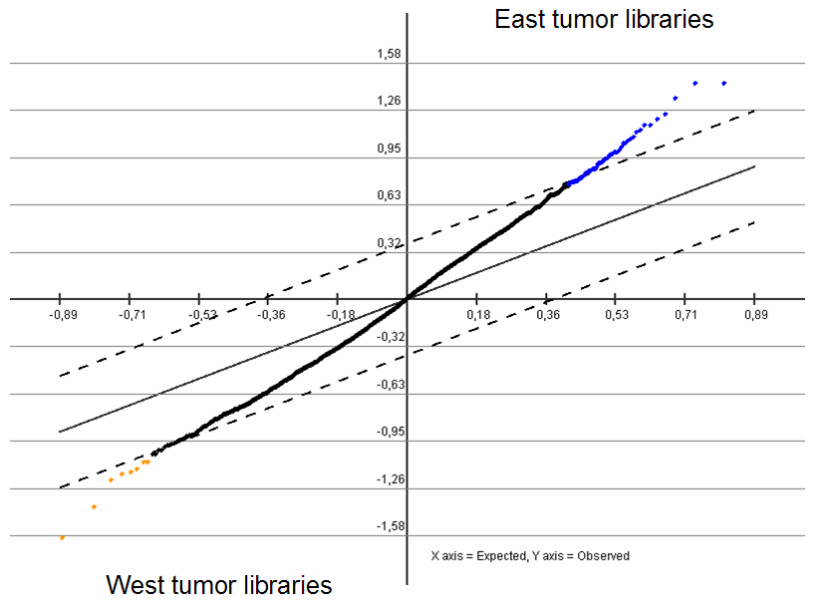
Serial Analysis for Microarray of East and West gastric carcinoma SAGE libraries. To the left and shown in orange color, the significant tags with higher expression in the West tumor libraries; to the right and shown in blue color, the significant tags with higher expression in the East tumor libraries.

Since the tumor side of the COA shows 2 groups, one containing all the East libraries and the other containing all the West libraries, we searched for discriminatory elements between both tumors libraries. Thus, a new selection process to find tags that were consistently expressed in "*all East tumors*" vs. "*all West tumors*" resulted in 3,952 tags. Another Support Tree using the Pearson Correlation and Average Linkage was performed. As shown in Fig. 3, the tree shows an organized structure with a high confidence degree in their branches (90%–100% support), given by the great number of discriminatory elements (tags) with distinctive families and subfamilies (the Additional File 3 shows the full dendrogram). There are two main clusters, one contains all West libraries and the other contains all East libraries. The West cluster contains two distinctive subclusters, the first contains the 3 microdissected libraries (CGAP_MD_HG7, CGAP_MD_HS29 and CGAP_MD_G329) and the second includes primary tumors (GSM757 and GSM2385) and xenografts (GSM758 and GSM14760). The East cluster contains a central pair (GSM8505 and GSM8867 libraries) that comes from histological well differentiated tumors and a third library (GSM7800) that comes from a histological poorly differentiated tumor. Next, to identify SAGE tags differentially expressed between the West and the East tumor libraries, we performed a SAM using the same criteria mentioned above. This approach revealed 54 tags differentially expressed (Fig. 4). Among these, 8 tags were up-regulated in the West tumors and 46 tags were up-regulated in the East tumors.

### Mapping SAGE tags to genes

**Figure 5:**
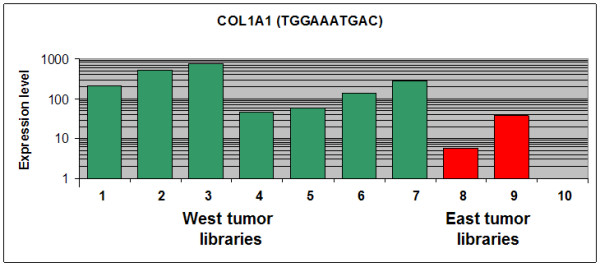
Expression levels of COL1A1 associated tag (TGGAAATGAC) in tumor libraries. Bars 1–7 correspond to all West tumor libraries (CGAP_MD_HG7, CGAP_MD_HS29, CGAP_MD_G329, GSM757, GSM758, GSM14760, GSM2385 and bars 8–10 correspond to all East tumor libraries (GSM7800, GSM8505, GSM8867). The tag normalized expression level appears in the CGAP format value (Tags per 200,000) plotted in a logarithmic scale.

For mapping differentially expressed SAGE tags to genes we used the CGAP-SAGE Genie and/or TAGmapper resources. Among the 90 tags differentially expressed between normal and tumor libraries, only 53 tags were successfully assignment to specific genes (Table S1 and Table S2 [Additional files 4 &5]). Genes like GIF, CPA2, DRD5, CLIC6, ATP4A, LIPF, GKN1 and PGA5 appear among the most repressed genes, while TRAPPC5, KRT7, MTHFD1, TMBIM1, PDIA3 and PPGB genes appear among the overexpressed genes. On the other side, among the 54 tags differentially expressed between the West and the East tumor libraries only 15 tags where successfully associated to specific genes (Table 1). FatiGO+ analysis showed that tumor libraries had significantly more expressed genes related to "cell organization and biogenesis" (GO:0016043), KRT7, PDIA3, PPGB and TRAPPC5 (p = 0.005); and "ligase activity" (GO:0016874), UBE2S and MTHFD1 (p = 0.028) than normal libraries,. The same comparison revealed significantly less expressed genes related to "integral to membrane" (GO:0016021), ADORA1, UGT2B15, DRD5, SYNE2, ATP5J2, KCNE2, ATP4A, KDR, PTGER3 and PPAP2B (p = 0.016). On the other hand, comparison of genes differentially expressed between the West and the East tumor libraries showed that the West tumors had significantly more expressed genes related to "ectoderm development" (GO:0007398) (COL1A1 shown on Fig. 5, also KLK10, KRT17, EMP1, and CCDC12) (p = 0.018). However, the East tumors had near significant more expressed genes related to "cellular metabolism" (GO: 0044237) PDGFRA, MAPK13, MECR, AKR1C2, RPL13, HLX1 and ADH4 (p = 0.066). Since at least two of these "ectoderm development" genes (COL1A1 and KLK10) have been found up-regulated in advanced gastric carcinoma [9,15] our findings might suggest more aggressiveness of the West tumors.

**Table 1:** The significant tags with higher expression by Significant Analysis for Microarray between the West and the East tumor SAGE libraries. Only the tags that were successfully associated with a specific gene are shown. The tags are sorted in a significance descending order, first the tags highly expressed in the East and then those highly expressed in the West.

**Tags**	**Gene Symbol**	**Protein Name**	**N° of West libraries where present**	**West tumor average (Tags per 200,000)**	**N° of East libraries where present**	**East tumor average (Tags per 200,000)**
TGATTGGTGG	PDGFRA	Platelet-derived growth factor receptor, alpha polypeptide	3	1.88	3	115.05
GGCTGGGTTT	HLX1	H2.0-like homeo box 1 (Drosophila)	2	1.04	3	59.13
TCCGTCCGGA	RPL13	Ribosomal protein L13	3	1.36	3	39.56
ATCTGGAGCA	ADH1C	Alcohol dehydrogenase 1C (class I), gamma polypeptide	3	5.99	3	294.91
TGCTCCTACC	FCGBP	Fc fragment of IgG binding protein	4	4.91	3	111.10
TACCCTGGAA	ADH4	Alcohol dehydrogenase 4 (class II), pi polypeptide	3	3.35	3	56.30
AGGTCTGCCA	AKR1C2	Aldo-keto reductase family 1, member C2 (dihydrodiol dehydrogenase 2; bile acid binding protein; 3-alpha hydroxysteroid dehydrogenase, type III)	3	1.53	3	38.50
GCACCACCGG	MAPK13	Mitogen-activated protein kinase 13	0	0	3	10.62
GGAGGGGAGG	MECR	Mitochondrial trans-2-enoyl-CoA reductase	1	0.55	3	15.72
CTTCCTTGCC	KRT17	Keratin 17	7	220.64	0	0
TAATTTGCAT	EMP1	Epithelial membrane protein 1	7	43.26	0	0
TAAGGCTTAA	KLK10	Kallikrein 10	7	20.35	0	0
TGGAAATGAC	COL1A1	Collagen, type I, alpha 1	7	294.99	2	14.36
TGGATGTACA	CCDC12	Coiled-coil domain containing 12	7	21.69	0	0

### Validation of genes differentially expressed between East and West tumor SAGE libraries

**Figure 6:**
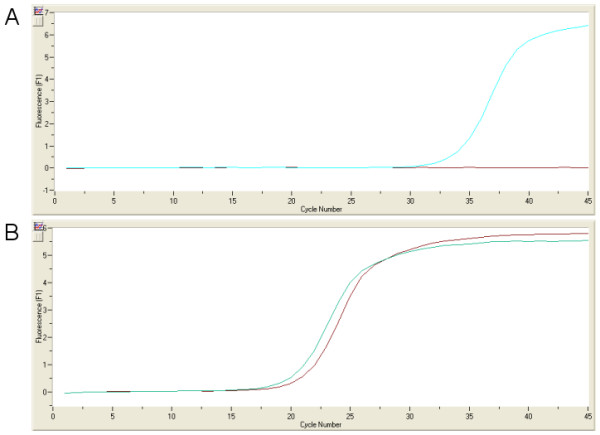
Amplification of PDGFRA (A) and RPL13 (B) mRNA by qRT-PCR. In (A) blue line is the East cell line (MKN45) and red line is the West cell line (N87). In (B) blue line is the East cell line (MKN45) and red line is the West cell line (AGS). Both genes are over-expressed in the East (MKN45) cell line.

To validated our SAGE data analysis two genes significantly more expressed in the East tumors (PDGFRA and RPL13) were further studied in three cell lines, two from the West (AGS and N87) and one from the East (MKN45). qRT-PCR shows a ratio of 825 for PDFGR (MKN45/N87) and 4.68 for RPL13 (MKN45/AGS) (Fig. 6). Thus, these data confirms the observed difference in gene expression in SAGE tumor libraries. Interestingly, the magnitudes of gene expression differences in cell lines were similar to that of in SAGE tumor libraries.

## Discussion

Our results, based on two non-supervised analyses, COA and Support Tree, are highly suggestive of a different expression profile of tumor SAGE libraries, along with differences between normal and tumor samples. These differences in expression levels might have an influence on the recognized better survival of the East patients in comparison to the West. Both, COA and Support Tree show two clusters (microdissected and non-microdissected samples) mixed indistinctly, suggesting that the heterogeneity of a normal sample is not reduced by the microdissection. This might be explained by multiple cell activities of the normal cells compared with tumor cells [16]. However among tumor libraries, a tight grouping of microdissected tumors was found. These findings suggest that the increase of the purity of the sample improves the homogeneity of the results. The neighborhood of the xenografts also points to an increase in homogeneity but differ from the microdissected tumor samples since they group in different subclusters. This difference is probably due to subtle changes in the transcriptomes given by a different genetic environment, such as the microenvironment given by surrounding animal tissue [17]. On the other hand, the non-microdissected libraries were found more scattered in the COA analysis, probably because of sample contamination and heterogeneity.

The FatiGO+ results show that the tumor cells are characterized by up-regulation of genes related to cell organization, biogenesis and cell proliferation, and a down-regulation of genes related to cell-to-cell communication. After searching for specific differences between the West and the East tumor libraries, we found that the most significantly different tags have a higher expression in the East compared with the West tumors. Thus, it seems that the average expression level of the West samples falls more than the East samples, probably because of a wider gene repression.

Of the 5 genes identified with significant higher expression in the West libraries at least two (COL1A1 and KLK10) have been associated with invasiveness and disease progression [9,15]. COL1A1 has been reported associated with more advanced tumor stage in 46 gastric carcinoma cases [9]. KLK10 has been reported up-regulated in gastric as well as colorectal carcinomas and associated with invasion and more advanced clinical stage for both types of tumors [15]. In addition KRT17 has been found up-regulated in human esophageal squamous cell carcinoma (ESCC) and associated to invasiveness [18]. Another gene, EMP1 has been associated to highly proliferative cell types in mouse brain tumors [19]. Only CCDC12 gene does not have available clinical data and also lacks GO annotations. The qRT-PCR analysis on cell lines confirmed the SAGE results and validated the over-expression of PDFGR and RPL13 in the East tumor libraries.

In summary here we report that the predominant up-regulation of invasive and metastatic genes in the West tumor libraries might result in a more malignant disease with a poorer survival. Taken together these findings might suggest that that differentially expressed genes might contribute to explain the observed differences observed in the outcome of gastric carcinoma between the East and the West. Finally, our analysis is an example of how computational biology can effectively assist biomedical researchers in identifying the molecular mechanisms of disease [6].

## Authors' contributions

FJO carried out the *in silico *analysis of SAGE databases, performed the bioinformatics analysis and drafted the manuscript.

CV participated in cell cultures, RNA extraction and carried out the RealTime PCR assays and drafted the manuscript.

FA carried out the cell culture, performed RNA extractions for the RealTime PCR assays and drafted the manuscript.

ES carried out the RealTime PCR assays.

NO participated in SAGE construction and SAGE database analysis.

WY participated in SAGE construction and SAGE database analysis and drafted the manuscript.

AHC conceived the study, participated in its design, performed the evaluation of results and drafted the manuscript.

All authors read and approved the final manuscript.

## Supplementary Material

Additional File 1 Correspondence Analysis of normal and tumor SAGE libraries of the stomach in 3 dimensions. The data provided represent the three-dimensional plot where the green dots represent all the normal libraries, the blue dots are the East tumor libraries, and the red, orange and yellow dots are West tumor libraries, microdissected, xenograft and bulk respectively. The X-axis is grey, the Y-axis is blue, and the Z-axis is pink. The figure is slightly rotated to the right and down to better show the tumor libraries position in the plot 3-D space. Click here for file

Additional File 2 Complete figure of Support Clustering Analysis of normal and tumor SAGE libraries of the stomach. The figure provided represent normal libraries CGAP_MD_13S, GSM784, CGAP_MD_14S, GSM14780 (lines 1–4), West tumor libraries CGAP_MD_HG7, CGAP_MD_HS29, CGAP_MD_G329, GSM757, GSM758, GSM14760, GSM2385 (lines 5–11) and East tumor libraries GSM7800, GSM8505, and GSM8867 (lanes 12–14). Click here for file

Additional File 3 Complete figure of Support Clustering Analysis of West and East tumor SAGE libraries of the stomach. The figure provided represent West tumor libraries (CGAP_MD_HG7, CGAP_MD_HS29, CGAP_MD_G329, GSM757, GSM758, GSM14760, GSM2385) (lanes 1–7) and East tumor libraries (GSM7800, GSM8505, and GSM8867) (lanes 8–10). Click here for file

Additional File 4 **Table S1.** The significant tags with higher expression in Normal by Significant Analysis for Microarray between Normal and Tumor SAGE libraries. Only the tags that were successfully associated with a specific gene are shown. The tags are sorted in a significance descending order. Click here for file

Additional File 5 **Table S2.** The significant tags with higher expression in Tumor by Significant Analysis for Microarray between Normal and Tumor SAGE libraries. Only the tags that were successfully associated with a specific gene are shown. The tags are sorted in a significance descending order. Click here for file
