# Supplementary figures and images for: In Silico analysis of Gastric carcinoma Serial Analysis of Gene Expression libraries reveals different profiles associated with ethnicity
Source: Mol Cancer. 2008 Feb 27;7:22. doi: 10.1186/1476-4598-7-22 (PMC2323622; doi:10.1186/1476-4598-7-22)

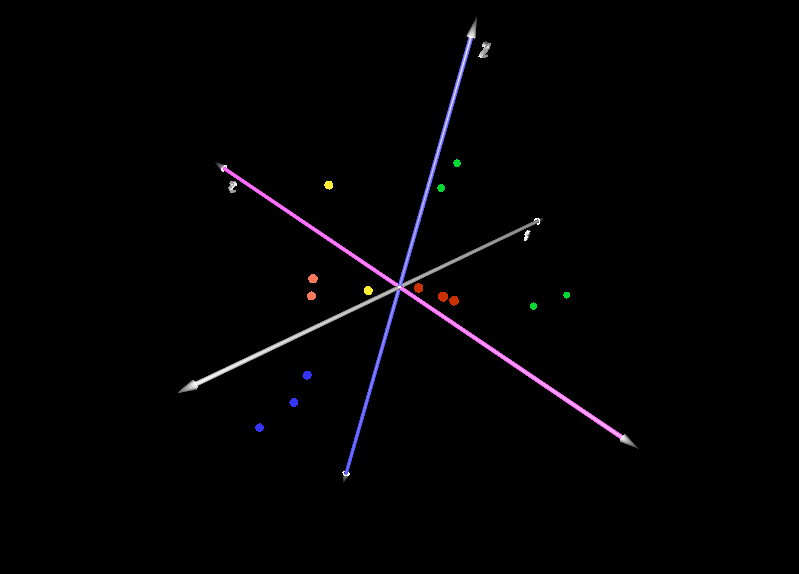

Supplement: Additional File 1 — Correspondence Analysis of normal and tumor SAGE libraries of the stomach in 3 dimensions. The data provided represent the three-dimensional plot where the green dots represent all the normal libraries, the blue dots are the East tumor libraries, and the red, orange and yellow dots are West tumor libraries, microdissected, xenograft and bulk respectively. The X-axis is grey, the Y-axis is blue, and the Z-axis is pink. The figure is slightly rotated to the right and down to better show the tumor libraries position in the plot 3-D space. [file 1476-4598-7-22-S1.png]

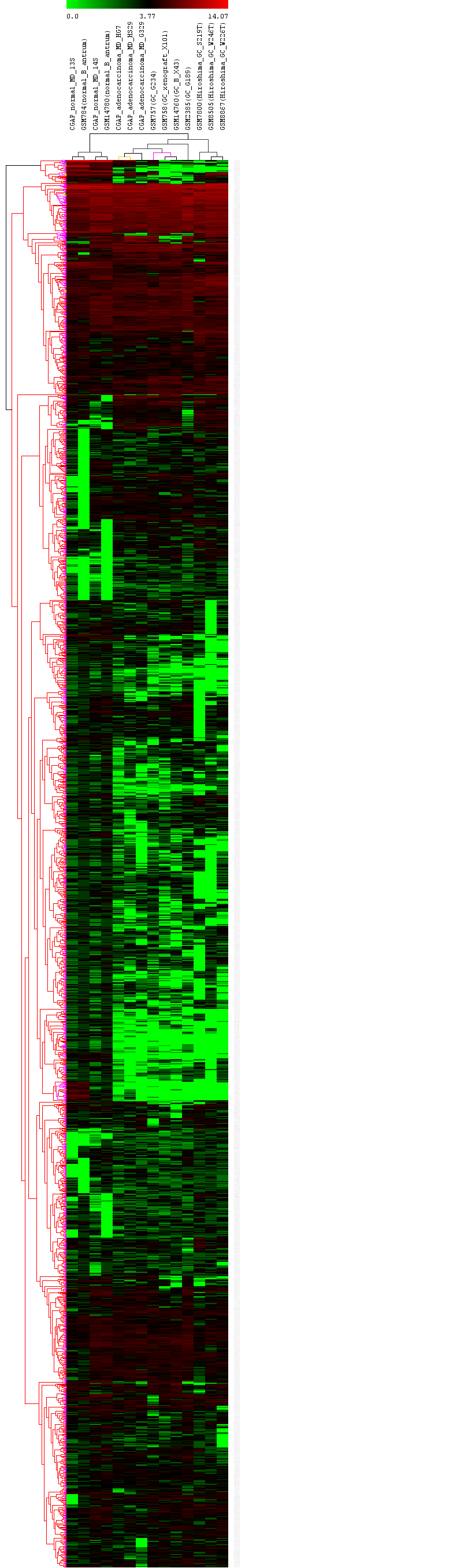

Supplement: Additional File 2 — Complete figure of Support Clustering Analysis of normal and tumor SAGE libraries of the stomach. The figure provided represent normal libraries CGAP_MD_13S, GSM784, CGAP_MD_14S, GSM14780 (lines 1–4), West tumor libraries CGAP_MD_HG7, CGAP_MD_HS29, CGAP_MD_G329, GSM757, GSM758, GSM14760, GSM2385 (lines 5–11) and East tumor libraries GSM7800, GSM8505, and GSM8867 (lanes 12–14). [file 1476-4598-7-22-S2.png]

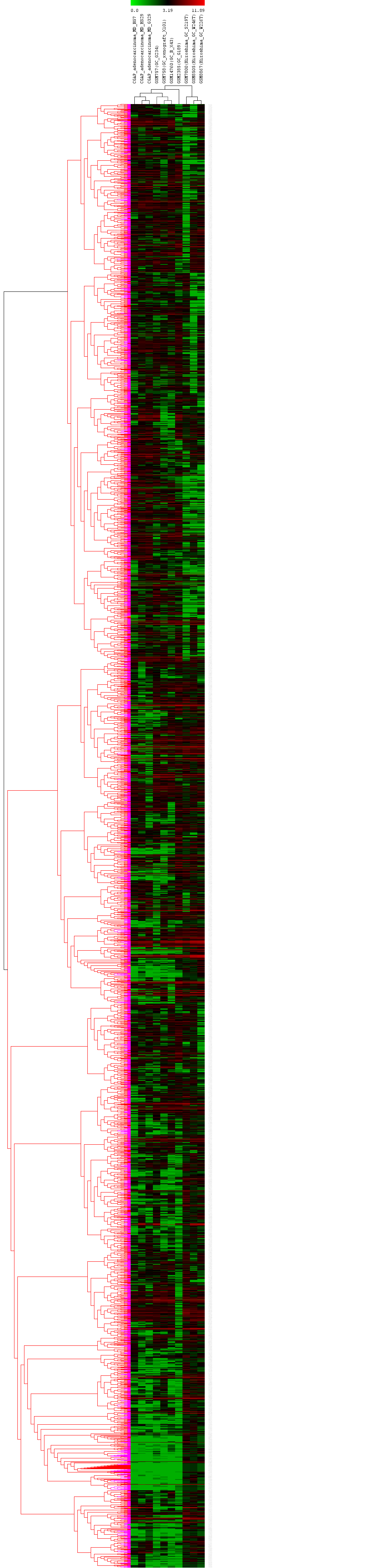

Supplement: Additional File 3 — Complete figure of Support Clustering Analysis of West and East tumor SAGE libraries of the stomach. The figure provided represent West tumor libraries (CGAP_MD_HG7, CGAP_MD_HS29, CGAP_MD_G329, GSM757, GSM758, GSM14760, GSM2385) (lanes 1–7) and East tumor libraries (GSM7800, GSM8505, and GSM8867) (lanes 8–10). [file 1476-4598-7-22-S3.png]
