# Supplementary material for: In Silico analysis of Gastric carcinoma Serial Analysis of Gene Expression libraries reveals different profiles associated with ethnicity
Source: Mol Cancer. 2008 Feb 27;7:22. doi: 10.1186/1476-4598-7-22 (PMC2323622; doi:10.1186/1476-4598-7-22)
Supplement: Additional File 4 — Table S1. The significant tags with higher expression in Normal by Significant Analysis for Microarray between Normal and Tumor SAGE libraries. Only the tags that were successfully associated with a specific gene are shown. The tags are sorted in a significance descending order. [file 1476-4598-7-22-S4.doc]

**Table S1**. The significant tags with higher expression in Normal by Significant Analysis for Microarray between Normal and Tumor SAGE libraries**.** Only the tags that were successfully associated with a specific gene are shown. The tags are sorted in a significance descending order.

| **Tags** | **Gene Symbol** | **Protein Name** | **Nº of Normal libraries where present** | **Normal average (Tags per 200,000)** | **Nº of Tumor libraries where present** | **Tumor average (Tags per 200,000)** |
| --- | --- | --- | --- | --- | --- | --- |
| GAGAACCACT | GIF | Gastric intrinsic factor (vitamin B synthesis) | 4 | 103.22 | 0 | 0 |
| TGCGAGACCA | CPA2 | Carboxypeptidase A2 (pancreatic) | 4 | 27.97 | 0 | 0 |
| TGTTTCTGTG | DRD5 | Dopamine receptor D5 | 4 | 22.93 | 0 | 0 |
| AGTGTGGAGT | KCNE2 | Potassium voltage-gated channel, Isk-related family, member 2 | 4 | 27.47 | 0 | 0 |
| TCGCAATTAC | REP15 | Rab15 effector protein | 4 | 18.81 | 0 | 0 |
| CAGTGCCTCT | ATP5J2 | ATP synthase, H+ transporting, mitochondrial F0 complex, subunit f, isoform 2 | 4 | 64.27 | 1 | 0.34 |
| TTTAGGATGA | GDDR | Blottin | 4 | 530.55 | 3 | 1.98 |
| ATAAATGATT | CLIC6 | Chloride intracellular channel 6 | 4 | 24.94 | 1 | 0.30 |
| CTGACTGTGC | ATP4A | ATPase, H+ K+ exchanging, alpha polypeptide | 4 | 455.02 | 3 | 3.48 |
| AATGTACCAA | LIPF | Lipase, gastric | 4 | 71.53 | 1 | 0.21 |
| CAGTGTTTCT | GOLGA3 | Golgi autoantigen, golgin subfamily a, 3 | 4 | 39.92 | 1 | 0.34 |
| ACCAAGTCAG | PTGER3 | Prostaglandin E receptor 3 (subtype EP3) | 4 | 10.46 | 0 | 0 |
| GAGATTATGT | KCNE2 | Potassium voltage-gated channel, Isk-related family, member 2 | 4 | 70.68 | 3 | 1.57 |
| TCATTCTGAA | GKN1 | Gastrokine 1 | 4 | 3925.18 | 4 | 15.88 |
| CAGCGCTTCT | PCSK5 | Proprotein convertase subtilisin/kexin type 5 | 4 | 54.16 | 0 | 0 |
| ACCTCCCCAC | CYP2B7P1 | Cytochrome P450, family 2, subfamily B, polypeptide 7 pseudogene 1 | 4 | 54.52 | 1 | 0.21 |
| TATTTAGCAA | GIF | Gastric intrinsic factor (vitamin B synthesis) | 4 | 29.06 | 1 | 0.34 |
| TTGCCCCTAC | CHIA | Chitinase, acidic | 4 | 252.24 | 2 | 2.13 |
| AACCTCCCCG | IGHG1 | Immunoglobulin heavy constant gamma 1 (G1m marker) | 4 | 45.74 | 0 | 0 |
| AACCTCCCCC | PGA5 | Pepsinogen 5, group I (pepsinogen A) | 4 | 134.53 | 2 | 0.77 |
| TCATTCCGAA | ETS2 | V-ets erythroblastosis virus E26 oncogene homolog 2 (avian) | 4 | 13.30 | 0 | 0 |
| CAGTGTTCTT | KDR | Kinase insert domain receptor (a type III receptor tyrosine kinase) | 4 | 31.29 | 2 | 0.55 |
| GATACGAGGT | GDDR | Blottin | 4 | 21.05 | 0 | 0 |
| AAATCCTGGG | TFF2 | Trefoil factor 2 (spasmolytic protein 1) | 4 | 1199.31 | 8 | 19.11 |
| TCTATAGCTG | ESRRG | Estrogen related receptor gamma | 4 | 16.83 | 1 | 0.34 |
| AACGTCCCCA | PGA5 | Pepsinogen 5, group I (pepsinogen A) | 4 | 1082.17 | 5 | 9.84 |
| TTTGGTACAT | MFSD4 | Major facilitator superfamily domain containing 4 | 4 | 18.06 | 2 | 0.76 |
| TATCTAAACT | RAB27A | RAB27A, member RAS oncogene family | 4 | 26.03 | 4 | 1.55 |
| ATCTGAAGCA | PCSK1N | Proprotein convertase subtilisin/kexin type 1 inhibitor | 4 | 21.30 | 2 | 1.07 |
| AACCTCCCCA | PGA5 | Pepsinogen 5, group I (pepsinogen A) | 4 | 9130.15 | 8 | 118.55 |
| TAATAAAATT | CPLX4 | Complexin 4 | 4 | 17.37 | 3 | 1.00 |
| GCAGGCTCCA | GHRL | Ghrelin/obestatin preprohormone | 4 | 68.01 | 3 | 1.41 |
| GAAACAACTT | UGT2B15 | UDP glucuronosyltransferase 2 family, polypeptide B15 | 4 | 23.41 | 2 | 0.76 |
| CACCTCCCCA | ADORA1 | Adenosine A1 receptor | 4 | 99.74 | 2 | 1.11 |
| ATGTATGTGT | ENAH | Enabled homolog (Drosophila) | 4 | 18.23 | 2 | 0.70 |
| CAGTGATTCT | SYNE2 | Spectrin repeat containing, nuclear envelope 2 | 4 | 54.46 | 2 | 0.73 |
| AGTGCTCTTC | PGC | Progastricsin (pepsinogen C) | 4 | 833.55 | 8 | 13.25 |
| CAGTGCTTAA | YWHAQ | Tyrosine 3-monooxygenase/tryptophan 5-monooxygenase activation protein, theta polypeptide | 4 | 30.63 | 0 | 0 |
| TTTTTCAAGA | UNQ473 | DMC | 4 | 41.59 | 6 | 2.46 |
| ATGTAGGTGC | PPAP2B | Phosphatidic acid phosphatase type 2B | 4 | 38.00 | 5 | 2.94 |
| CACCCCTGAT | CKB | Creatine kinase, brain | 4 | 95.58 | 6 | 9.03 |
| GCCCAGCATT | PSCA | Prostate stem cell antigen | 4 | 140.12 | 5 | 12.70 |
