# Supplementary material for: In Silico analysis of Gastric carcinoma Serial Analysis of Gene Expression libraries reveals different profiles associated with ethnicity
Source: Mol Cancer. 2008 Feb 27;7:22. doi: 10.1186/1476-4598-7-22 (PMC2323622; doi:10.1186/1476-4598-7-22)
Supplement: Additional File 5 — Table S2. The significant tags with higher expression in Tumor by Significant Analysis for Microarray between Normal and Tumor SAGE libraries. Only the tags that were successfully associated with a specific gene are shown. The tags are sorted in a significance descending order. [file 1476-4598-7-22-S5.doc]

**Table S2**. The significant tags with higher expression in Tumor by Significant Analysis for Microarray between Normal and Tumor SAGE libraries**.** Only the tags that were successfully associated with a specific gene are shown. The tags are sorted in a significance descending order.

| **Tags** | **Gene Symbol** | **Protein Name** | **Nº of Normal libraries where present** | **Normal average (Tags per 200,000)** | **Nº of Tumor libraries where present** | **Tumor average (Tags per 200,000)** |
| --- | --- | --- | --- | --- | --- | --- |
| ATCAAGTTCG | TRAPPC5 | Trafficking protein particle complex 5 | 0 | 0 | 10 | 12.85 |
| CCTGGTCCCA | KRT7 | Keratin 7 | 1 | 0,68 | 10 | 121,82 |
| TCTGTTTACT | MTHFD1 | Methylenetetrahydrofolate dehydrogenase (NADP+ dependent) 1, methenyltetrahydrofolate cyclohydrolase, formyltetrahydrofolate synthetase | 0 | 0 | 10 | 13.11 |
| TGGCCATCTG | TMBIM1 | Transmembrane BAX inhibitor motif containing 1 | 1 | 1,09 | 10 | 40,79 |
| ATGCGGGAGA | TMEM54 | Transmembrane protein 54 | 1 | 0,68 | 10 | 24,22 |
| CAGGAGGAGT | PDIA3 | Protein disulfide isomerase | 1 | 2,18 | 10 | 44,47 |
| CTGGCGAGCG | UBE2S | Ubiquitin-conjugating enzyme E2S | 1 | 0.68 | 10 | 15.56 |
| GGCTCCTGGC | ITGB4BP | Integrin beta 4 binding protein | 1 | 2.18 | 10 | 36.22 |
| TGGGTGGGGG | LOC339123 | Hypothetical LOC339123 | 1 | 0.68 | 10 | 15.44 |
| TTCTCCCGCT | PPGB | Protective protein for beta-galactosidase (galactosialidosis) | 0 | 0 | 10 | 13.60 |
| ACGTGGTGAT | C19orf53 | Chromosome 19 open reading frame 53 | 0 | 0 | 10 | 12.38 |
